# Rapid Response of Mediastinal Lymphoma to Corticosteroids During Diagnostic Evaluation: A Clinical Case Report

**DOI:** 10.7759/cureus.59103

**Published:** 2024-04-26

**Authors:** K K Athish, Guruprasad T J, Spurthy Padmanabha, Harshitha K R

**Affiliations:** 1 Internal Medicine, Sri Devaraj Urs Academy of Higher Education and Research, Kolar, IND; 2 Respiratory Medicine, Sri Devaraj Urs Academy of Higher Education and Research, Kolar, IND; 3 Pulmonology, Sri Devaraj Urs Academy of Higher Education and Research, Kolar, IND

**Keywords:** pericardial effusion, anterior mediastinal mass, pleural effusion, precursor t-cell acute lymphoblastic lymphoma, steroid responsive lymphoma

## Abstract

Here, we report a case of non-Hodgkin's lymphoma in a 21-year-old man who presented with symptoms of gastric discomfort, hematemesis, breathlessness, dry cough, chest pain, loss of appetite, and weight loss. He had a history of pleural effusion and was previously diagnosed with tuberculosis. Further investigations revealed a mediastinal mass. A biopsy confirmed non-Hodgkin's lymphoma and ruled out thymoma. The patient underwent therapeutic thoracentesis for symptomatic relief and was started on chemotherapy. The prognosis of T-cell lymphoblastic lymphoma (T-LBL) is generally poorer compared to B-cell lymphoblastic lymphoma (B-LBL). T-LBL commonly presents with a mediastinal mass and pleural effusion. Imaging techniques like computed tomography (CT) help evaluate the extent and characteristics of the tumor. Prognostic factors for T-LBL include age, pleural effusion, and extranodal involvement. Molecular characterization is important in determining prognosis and treatment options. 18F-FDG imaging can assist in determining the extent of the tumor, staging, and assessment of response to treatment. Overall, lymphoblastic lymphoma is a rare entity, and T-LBL accounts for a small percentage of all lymphomas. Before the start of definitive chemotherapy, during the evaluation, the patient was started on steroid therapy for symptomatic management, following which regression in the size of the mediastinal tumor was noted.

## Introduction

Lymphoblastic neoplasms comprise lymphoblastic lymphomas (LBLs) and lymphoblastic leukemias. These neoplasms are clonal proliferations of lymphoid progenitors primarily of T-cell or B-cell descent, with 70-80% originating from T-lymphoblasts and 20-25% from B-lymphoblasts, although the natural killer (NK) phenotype may occasionally be seen [1,2].

Mixed phenotypes, including myeloid/lymphoblastic origins (MPAL), are rare. Acute lymphoblastic leukemia (ALL) and LBL are considered to be on the same spectrum of disease according to recent WHO classifications, sharing clinical manifestations but are differentiated by the degree of involvement of peripheral blood and/or bone marrow. T-ALL is considered when 25% of lymphoblasts are in the bone marrow (BM) [2]. A tumor of immature T-cell precursors, T-cell lymphoblastic lymphoma (T-LLy), is aggressive, accounting for 25% to 30% of juvenile non-Hodgkin lymphomas. Studies on the genome and gene expression have revealed that T-LBL and T-ALL differ in their transcriptional and genetic makeup [3]. The present complaints are related to the anterior mediastinal mass and lymph nodes. The percentage of T-cell LBL patients who have bone marrow (BM) involvement is just 25-30%. Local recurrence is common, similar to the disease in bone marrow and the central nervous system [4]. When prednisolone was used alone for the treatment of acute lymphoblastic leukemia (ALL), early trials revealed that affected children experienced rapid clinical improvement and remission. Nevertheless, the remission lasted for a short duration, and its recurrence was unavoidable. Hence, multiple medication therapies, including cytotoxic drugs, were combined with steroids. According to more recent research, at conventionally equivalent dosages, dexamethasone is observed to be a more effective anti-leukemic medication than prednisolone [5]. With corticosteroids and appropriate multiagent induction chemotherapy, over 90% of patients have achieved complete remission by 1-2 months [6].

## Case presentation

**Figure 1:**
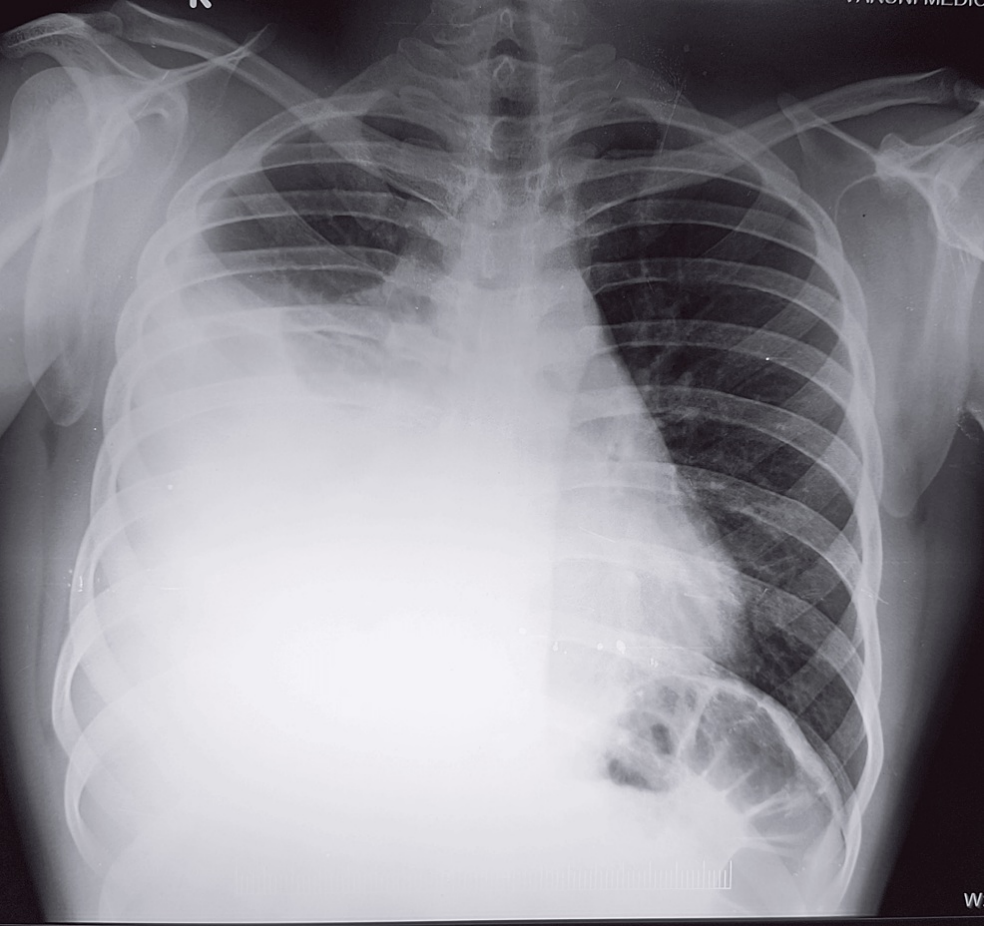
Initial chest X-rays revealed right costophrenic angle obliteration with a meniscus sign.

A 21-year-old man presented to an outside hospital with gastric discomfort for one week and an episode of hematemesis. He was admitted to a local hospital four weeks ago, and a posterior-anterior view of the chest X-ray revealed right costophrenic angle obliteration with a meniscus sign suggestive of a large right-sided pleural effusion (Figure 1). 

Diagnostic and therapeutic pleural fluid aspiration was performed, and analysis was suggestive of exudative effusion with high adenosine deaminase (ADA), following which he was initiated on anti-tubercular therapy (ATT). He had a negative medical history and was not on any medications before these complaints.

**Figure 2:**
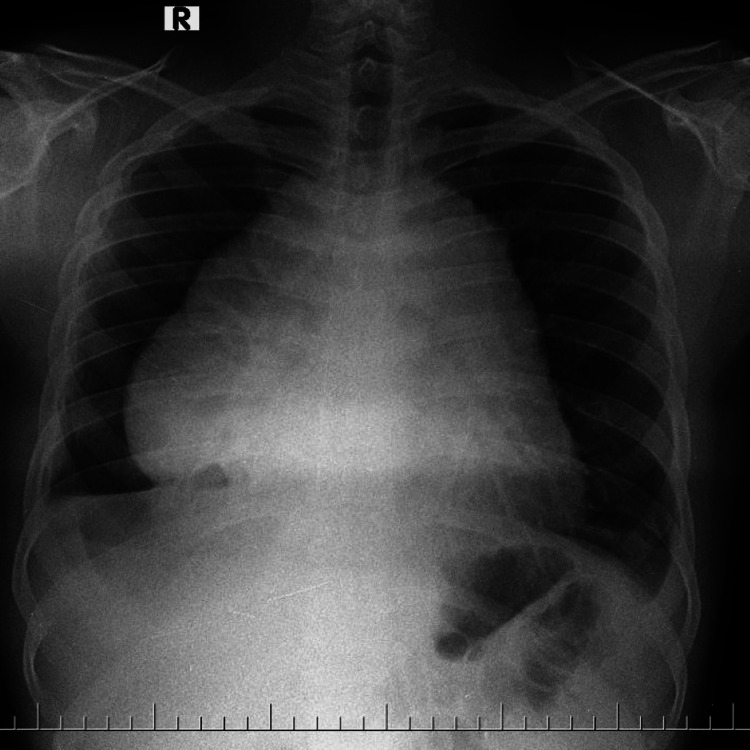
A post-thoracentesis chest radiograph revealed an expanded lung. Also, evidence of mediastinal widening with the hilum overlay sign and cardiomegaly was noted.

After three weeks, he presented to our hospital with exertional breathlessness, a dry cough, and non-radiating chest pain for three days. History of loss of appetite and significant weight loss history over six weeks (~12 kgs). There was no history of hoarseness of voice, stridor, dysphagia, fever, chills, night sweats, facial or arm swelling, headaches, hemoptysis, or vomiting. Vitals were: heart rate 113 beats/minute, respiratory rate 33 cycles/minute, blood pressure 100/72 mmHg, and oxygen saturation was 95% on room air. The patient was moderately built, conscious, and oriented to time, place, and person. Icterus was present. Pallor, cyanosis, clubbing, lymphadenopathy, and edema were absent. The respiratory system examination revealed dullness on percussion over the right mammary, infra-axillary and infrascapular regions, and d’espine sign were present. On auscultation, decreased breath sounds on the right side and normal vesicular breath sounds were heard on the left side. A cardiovascular system examination revealed muffled heart sounds. The abdomen examination was normal. A chest radiograph revealed mediastinal widening with hilum overlay and cardiomegaly (Figure 2).

**Figure 3:**
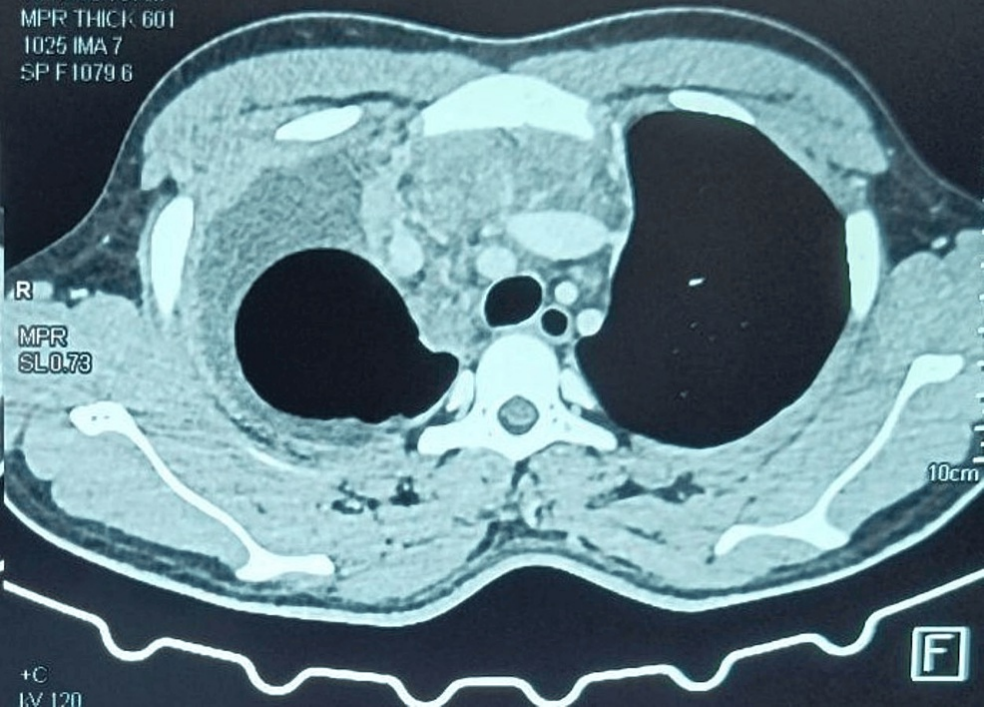
Contrast-enhanced computed tomography scan of the thorax: axial cut, mediastinal window showing multiple enhancing pre-tracheal, cardio-phrenic, prevascular, and aortopulmonary lymph nodes with adjacent right pleural effusion and lung collapse.

**Figure 4:**
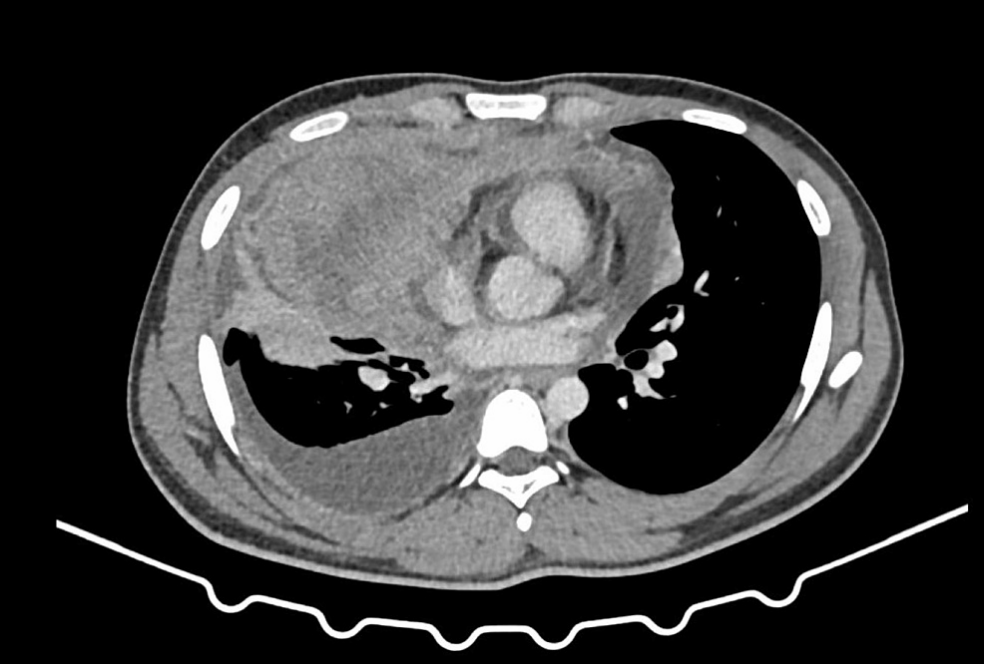
Contrast-enhanced computed tomography scan of the thorax: axial cut, mediastinal window showing a well-defined, heterogeneously enhancing soft tissue density lesion measuring ~ 7.85 x 16.5 x 10.0 cm (AP x TR x CC) noted in the anterior mediastinum predominantly on the right side with extensions to the left. Few non-enhancing areas are noted within the likely necrosis, with no calcifications noted.

**Figure 5:**
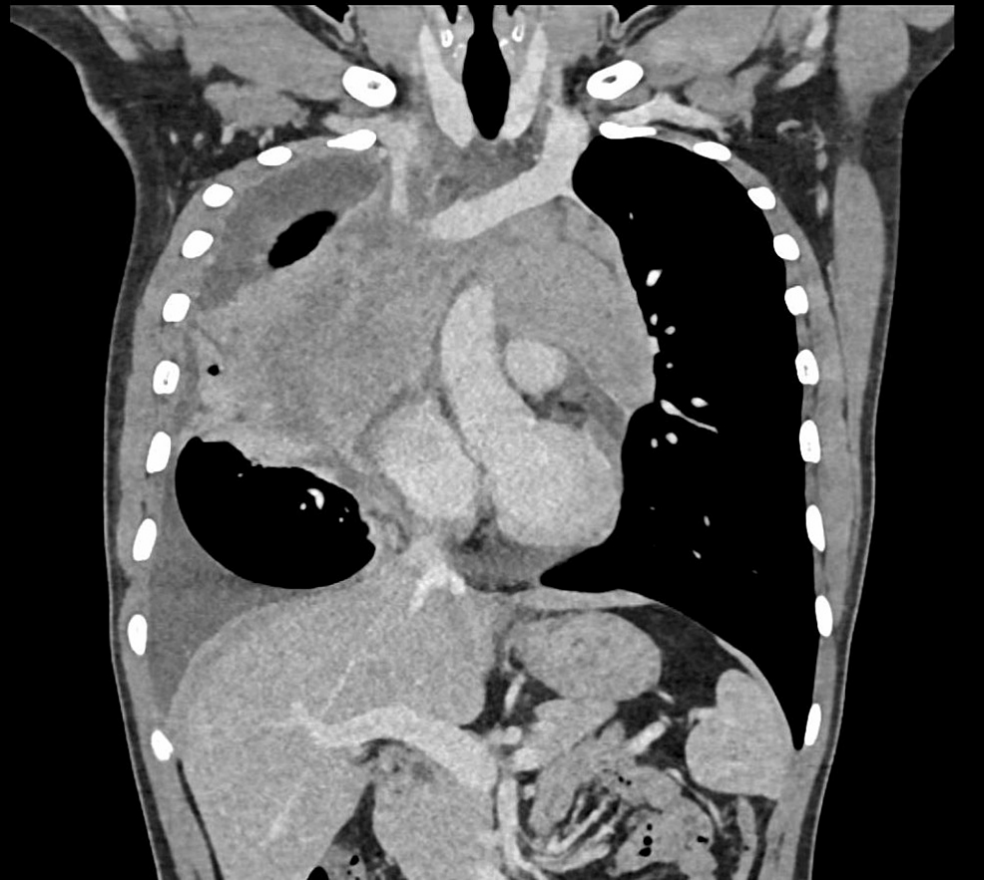
Contrast-enhanced computed tomography scan of the thorax-coronal section shows a lesion extending anterio-superiorly from the level of the sternoclavicular joint to the level of the T6 vertebral body inferiorly. The lesion extends into the aortopulmonary window and encases the arch of the aorta, ascending aorta, main pulmonary trunk, superior vena cava, and left brachiocephalic vein for an arc of 270 degrees. It encases the right and left main pulmonary arteries for an arc of 180 degrees and is also seen abutting the right and left upper lobe segmental bronchi and arteries, and bilateral superior and inferior pulmonary veins.

**Figure 6:**
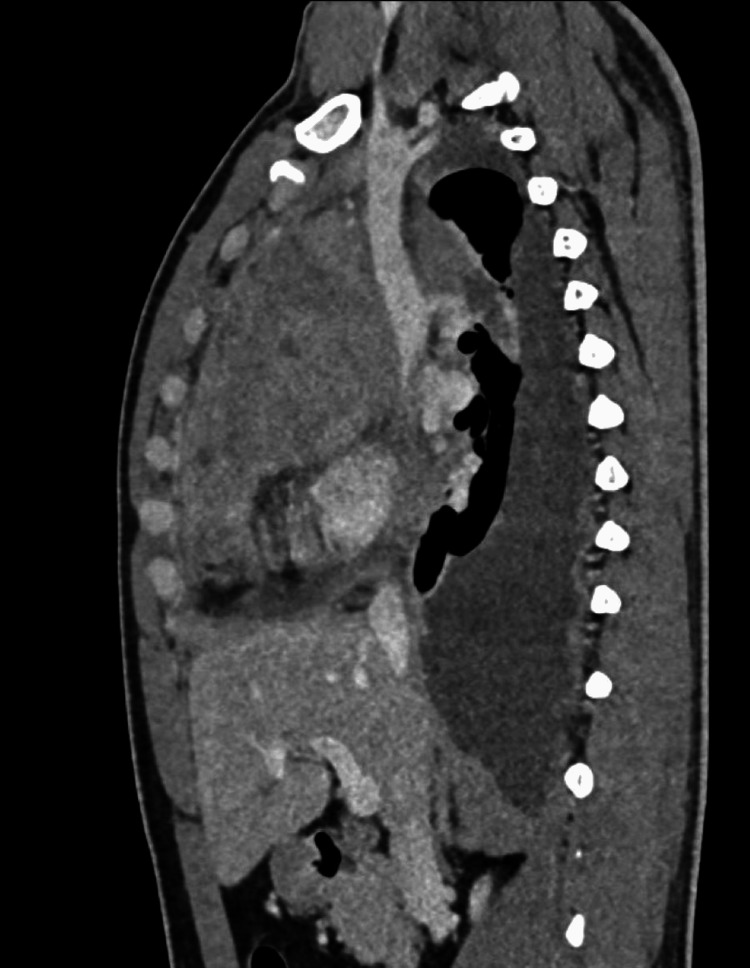
Contrast-enhanced computed tomography scan: sagittal section of mediastinal window showing an anterior mediastinal mass with right side pleural effusion.

2D ECHO revealed normal chamber dimensions with a pulmonary artery systolic pressure (PASP) of 28 mmHg, moderate pericardial effusion with large right-sided and minimal left-sided pleural effusion, and an ejection fraction of 60%. Routine investigations revealed hemoglobin of 15 g/dl, white blood cells of 8.2 T/mm3, and platelets of 438 T/mm3 with normal peripheral smear and serum electrolytes. Liver function tests were deranged, and ATT was withheld. Contrast-enhanced computed tomography (CECT) of the thorax (Figures 3-6) revealed a large mediastinal mass lesion abutting the pericardium with loss of fat planes. Laterally, the lesion was seen extending to the left lateral chest wall; however, there was no erosion of the ribs. Multiple heterogeneously enhancing pre-tracheal, cardio-phrenic, prevascular, and aortopulmonary window lymph nodes were noted, the largest measuring ~ 1.8 x 2.2 cm in the right cardio-phrenic region. Right moderate pleural effusion with collapse or consolidation of the underlying parenchyma was noted. Left lung parenchyma appeared normal with a normal broncho-vascular pattern.

**Figure 7:**
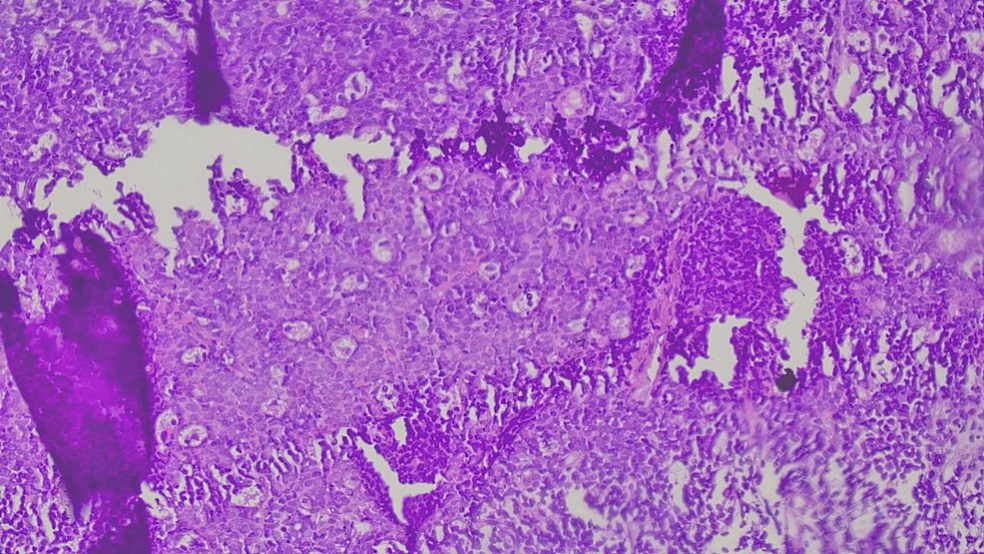
Histopathology image showing monotonous population of lymphocytes was arranged in sheets and singles, individual tumor cells were medium to large size with condensed chromatin.

**Figure 8:**
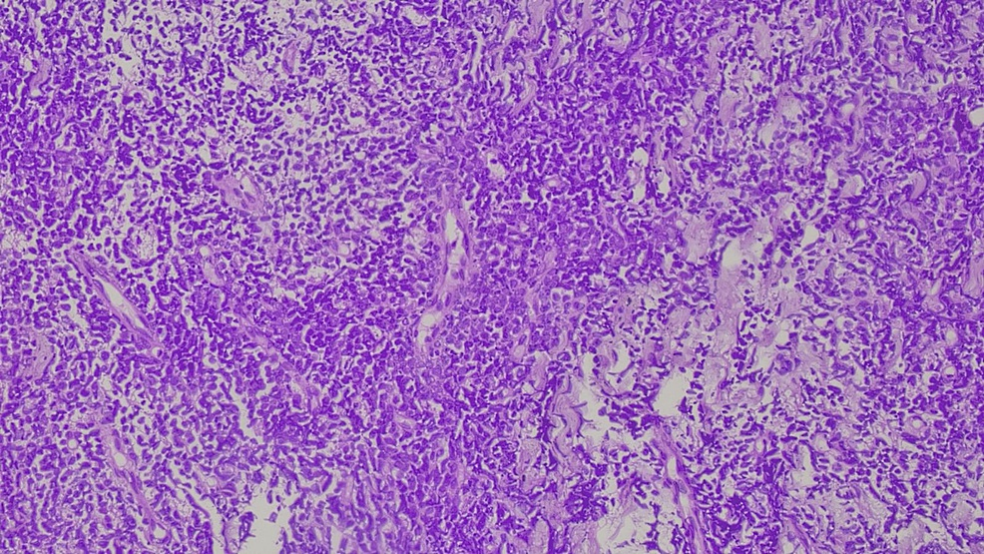
Histopathology image showing round to oval with abundant eosinophilic cytoplasm and prominent eosinophilic nucleus.

The differential diagnoses by CECT thorax were 1) malignant germ cell tumor, 2) invasive thymоma, and 3) lymphoma. A USG-guided biopsy was performed, and the microscopically monotonous population of lymphocytes was arranged in sheets and singles. Individual tumor cells were medium to large with condensed chromatin, round to oval with abundant eosinophilic cytoplasm and a prominent eosinophilic nucleus (Figures 7, 8). Based on the features, histopathologically, non-Hodgkin’s lymphoma and thymoma were the differentials. ATT was not restarted as there were no clinical indications. 

**Figure 9:**
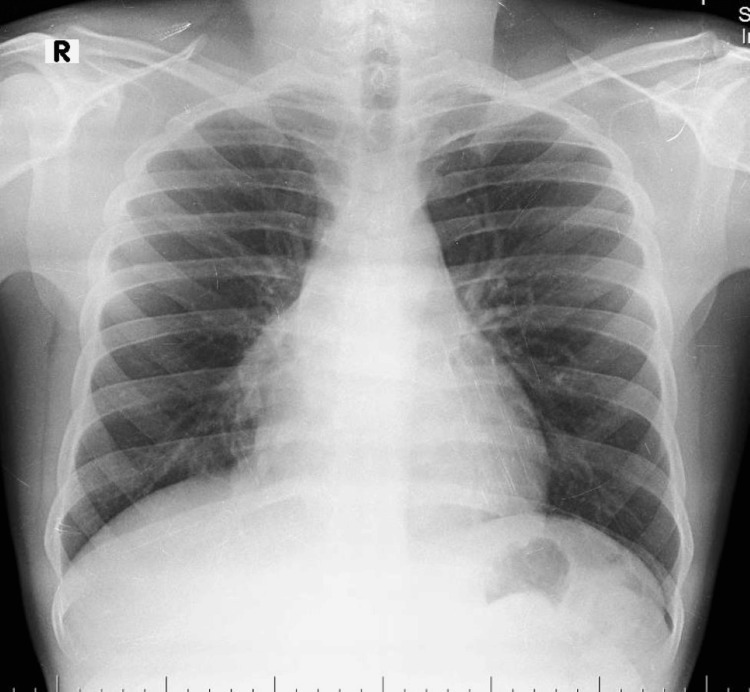
Chest radiograph following therapeutic thoracentesis and steroid therapy, a comparative reduction in the size of the mediastinal mass and resolution of the pleural effusion were noted.

Pleural fluid was positive for malignancy. Immunohistochemistry revealed lymphoblastic cells, which were positive for leukocyte common antigen (LCA), CD 7, TdT, and CD 99, with a Ki67 proliferation index of 90% and negative for CK, CD5, and CD 20. Positron emission tomography with FDG revealed a hypermetabolic homogeneously enhanced mass lesion in the anterior mediastinum measuring 7×6.5×7.1 cm SUV max 5.7 without enhancement elsewhere. More than a 50% reduction in tumor size was noted between the two consecutive CT studies, even before the initiation of definitive chemotherapy. The results of the bone marrow biopsy showed that flow cytometry was used for analysis, specifically using forward-side scatter and CD45-side scatter. The analysis revealed negative results for the following markers: CD45, HLA DR, CD34, TdT, CDla, CD3, CD4, CD7, CD99, CD8, CD5, CyCD3, CD19, CD10, CD79a, CD13, CD117, MPO, CD64, CD33, CD14, and CD43, though few markers were positive in tissue biopsy. No bone marrow involvement was thus confirmed. Before the initiation of definitive treatment, a chest radiograph (Figure 9) was performed following therapeutic thoracentesis and steroid therapy. A comparative reduction in the size of the mediastinal mass and resolution of the pleural effusion were noted. Hence, the patient was diagnosed with precursor T-cell acute lymphoblastic lymphoma. The patient was initiated on Inj Vincristine 2mg and Inj Daunorubicin 10mg with prednisolone for induction therapy. The patient tolerated the chemotherapy well.

## Discussion

Lymphoblastic lymphoma is a rare entity, which accounts for 8% of all lymphoid malignancies [7]. T-cell lymphoma accounts for nearly 2% of all lymphomas and is an uncommon variety of non-Hodgkin's lymphoma with an approximate estimate of 0.1/1 lakh inhabitants/year, with male predilection, especially observed among adolescents and young adults [7,8]. The clinical manifestations in patients with primary mediastinal mass, as per the literature, are chest pain, dullness, breathlessness, weight loss, cough, invasion of the recurrent laryngeal nerve or the superior laryngeal nerve causing hoarseness of voice, dysphagia, tracheal deviation, palpable cervical lymphadenopathy, superior vena cava syndrome, neurological signs like wasting of muscles, loss of muscle power, and exaggerated deep tendon reflexes [9,10]. The tumor expands aggressively in the anterior mediastinum and has the potential to invade the gonads, the bone marrow, and the central nervous system. In LBL patients, about 80% of the patients had a mediastinal mass, and 60% also had a pleural or pericardial effusion. Our patient is 21 years old and presented with bilateral pleural effusion, pericardial effusion, and a giant mediastinal mass. Generally, the T-phenotype relatively exhibits a poorer prognosis than the B-cell lineage [11].

Local lymph node enlargement is an occasional symptom of lymphoblast disease (LBL), often involving nearby tissues and organs. Other than invasion and concealment, these tumors typically lack any distinguishing characteristics. A significant giant mediastinal mass that occupies a wider mediastinum, which might be obtuse and convex relative to the lung, is frequently visible on a chest radiograph. On CT imaging, the tumor appears as a significant, irregular bulk with a large focus, as shown on the enhanced scan. CT scans can also show the extent of vascularization and the anatomical relationships between the mass and the mediastinal structure [12]. Examining the borders and internal characteristics of a lesion may help narrow down the range of potential differential diagnoses when the lesion's shape or location are similar to one another. Typically, malignant tumors exhibit invasive characteristics and asymmetrical borders. Internal characteristics, such as nodular staining and hyper- or hypovascularity, can also be seen using contrast tests. It is possible to distinguish between more obvious cases of calcification, hemorrhage, or fat deposition using CT results than between more prominent ones [13].

Prognostic factors like age, sex, fever or infection, splenomegaly, hepatomegaly, mediastinal mass, lymphadenopathy, initial platelet count (>100 × 109), leukocyte count (>30 × 10), LDH level, and immunophenotypic subtype do not significantly affect survival with T-LBL. Significantly lower overall survival was linked to the existence of pleural effusion and ≥2 extranodal involvement [11]. Prognostic predictors can help lower-risk individuals avoid overtreatment and the ensuing acute and long-term toxicities by identifying those who are most at risk of relapsing, which is frequently life-threatening. These patients would also benefit from the early introduction of innovative medicines. Since clinical criteria are not predictive in T-LBL, the focus has shifted to molecular characterization [2].

Although there is a broad range of genetic and molecular abnormalities found in T-lymphoblastic processes, several are consistently observed. These include rearrangements of the TCR gene locus, NOTCH1, CDKN2A, HOX gene group, and TAL1 (SCL). Fifty to seventy percent of cases have abnormal karyotypes [1].

A key component for the diagnosis and differential diagnosis of malignant lymphoma may be provided by 18-FDG imaging. With its increased sensitivity and specificity over traditional CT, 18F-FDG imaging can aid in diagnosis and treatment. Many lymphomas show high 18-FDG uptake because malignant lymphomas typically have higher cell densities than other types of malignancy. After several research articles were reviewed, it was determined that lymphoma histology plays a major role in determining the degree of FDG uptake, with the aggressive kind of disease typically showing higher uptake. The diagnosis and differential diagnosis of malignant lymphoma may greatly benefit from the use of 18F-FDG imaging [14].

Precursor T-cell LBL is an aggressive form of lymphoma with unfortunately high relapse rates, even though there is an excellent initial response and five-year disease-free survival rates of 60%-80%. [15] Treatment requires regimens similar to those employed in treating NHL, such as CHOP, for which response rates may vary from 55% to 95%. However, upon avid literature review, a hyper-CVAD regimen with Nelatabine is used in patients aged 18 and above with a response rate of 70% [16]. An augmented BFM regimen is also employed in patients over 40 years old. However, both of these regimens show significantly poor outcomes for early precursor T cell (EPTC) ALL compared to non-EPTC lymphomas, with an increased risk of relapse [17].

## Conclusions

Although lymphoma is common in clinical practice, it is often misdiagnosed, causing delayed treatment initiation and affecting patient outcomes as the disease progresses. In the above case, the diagnosis of lymphoma was initially missed due to a lack of suspicion of a mediastinal tumor based on chest radiograph findings, and the patient was clinically diagnosed with tuberculosis. Here, we discussed the imaging modality and ultrasound-guided needle biopsy with histopathological evaluation aiding in the diagnosis of non-Hodgkins lymphoma. However, we share our experience regarding the rapid reduction of tumor dimension with corticosteroid therapy during the evaluation period before arriving at a definite diagnosis.
